# Microparticles: biogenesis, characteristics and intervention therapy for cancers in preclinical and clinical research

**DOI:** 10.1186/s12951-022-01358-0

**Published:** 2022-04-13

**Authors:** Yan Hu, Yajie Sun, Chao Wan, Xiaomeng Dai, Shuhui Wu, Pui-Chi Lo, Jing Huang, Jonathan F. Lovell, Honglin Jin, Kunyu Yang

**Affiliations:** 1 Cancer Center, Union Hospital, Tongji Medical College, Huazhong University of Science and Technology, Wuhan, 430022 China; 2 Department of Medical Oncology, The First Affiliated Hospital, College of Medicine, Zhejiang University, Hangzhou, China; 3 Department of Biomedical Sciences, City University of Hong Kong, Tat Chee Avenue, Kowloon, Hong kong, China; 4 College of Biomedicine and Health and College of Life Science and Technology, Huazhong Agricultural University, Wuhan, 430070 China; 5 Department of Biomedical Engineering, University at Buffalo, State University of New York, Buffalo, NY 14260 USA

**Keywords:** Drug delivery, Extracellular vesicles, Microparticles, Cancer treatment, Immunotherapy

## Abstract

Extracellular vesicles (EVs), spherical biological vesicles, mainly contain nucleic acids, proteins, lipids and metabolites for biological information transfer between cells. Microparticles (MPs), a subtype of EVs, directly emerge from plasma membranes, and have gained interest in recent years. Specific cell stimulation conditions, such as ultraviolet and X-rays irradiation, can induce the release of MPs, which are endowed with unique antitumor functionalities, either for therapeutic vaccines or as direct antitumor agents. Moreover, the size of MPs (100–1000 nm) and their spherical structures surrounded by a lipid bilayer membrane allow MPs to function as delivery vectors for bioactive antitumor compounds, with favorable phamacokinetic behavior, immunostimulatory activity and biological function, without inherent carrier-specific toxic side effects. In this review, the mechanisms underlying MP biogenesis, factors that influence MP production, properties of MP membranes, size, composition and isolation methods of MPs are discussed. Additionally, the applications and mechanisms of action of MPs, as well as the main hurdles for their applications in cancer management, are introduced.

**Graphical Abstract:**

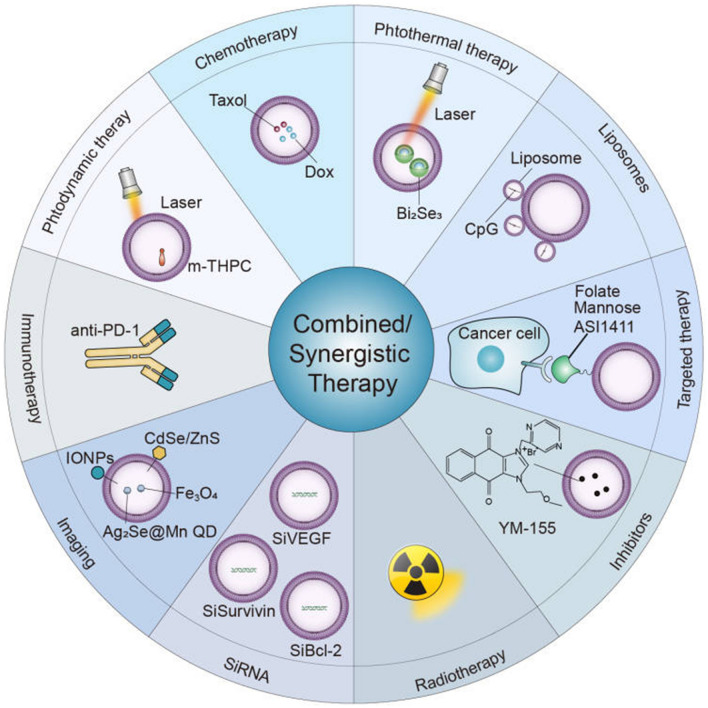

## Background

Extracellular vesicles (EVs), spherical biological particles with lipid bilayers, can be generated and released in large amounts from cells after exposure to various stimuli, such as hypoxia, hunger and oxidative stress [1]. As a means of communication between cells, EVs carry a variety of bioactive substances, including nucleic acids, proteins, lipids, and metabolites, and deliver them to target cells for information transmission through autocrine, paracrine, or endocrine pathways [2, 3].

According to their diameters and formation mechanisms, EVs are divided into three categories: exosomes (EXOs), microparticles (MPs) and apoptotic bodies. EXOs (30–100 nm) are secreted from an endocytic chamber called the multivesicular body. In contrast, MPs (100–1000 nm), which were first described as platelet “dust” in plasma in the 1960s [4], directly bud from the plasma membrane. Similarly, apoptotic bodies (1000–5000 nm) are also shed from the plasma membrane of apoptotic cells. Characteristic distinguishing features of EVs are listed in Table 1. Considering the natural and therefore biologically compatible membrane structure of EVs, research efforts have investigated EVs for drug delivery for disease treatment. Until now, EXOs have been the most widely studied, and there have been many reports on their formation and applications. However, recently, increasing focus has turned to MPs, which not only serve as therapeutic agents but also can be utilized as drug delivery vesicles [5]. While there EXOs have extensively in the literature for their roles as therapeutic and diagnostic tools for various diseases, less focus has been given to MPs. This review is aimed to assist those interested in the field in understanding MPs, for better directing investigations into fundamental mechanisms and new treatments for disease, ultimately leading to clinical transformations.

**Table 1:** Comparisons of EVs

Characteristic	EXOs	MPs	Apoptotic bodies
Origin	Multivesicular bodies	Plasma membrane	Plasma membrane
Size	30–100 nm	100–1000 nm	1000–5000 nm
Density	1.13–1.19 g/cm^3^	1.04–1.07 g/cm^3^	1.16–1.28 g/cm^3^
Sedimentation	≥ 100000 g	10000–20000 g	2000 g and various
Zeta potential	− 16.35 ~ − 11.85 mV	− 30 ~ − 10 mV	/
Appearance	Cup-shaped	Irregular-shaped	Irregular-shaped
Markers	TSG101, tetraspanins and Alix	Integrins, selectins and CD40 ligand	Histones, Annexin V

Herein, we first briefly introduce the mechanisms of MP biogenesis and factors that influence MP production, followed by a description of properties of MP membrane, size and internal composition and isolation methods of MPs. Subsequently, we review the applications of MPs as therapeutics agents and then discuss advantages and disadvantages of MPs as drug delivery system (DDS), in comparison to EXOs and artificial nanoparticles, followed by specific application and mechanisms of action of MPs in cancer. Finally, we discuss the potential and challenges for MPs in clinical translation.

## Biogenesis of MPs

### Mechanisms of MP biogenesis

**Fig. 1:**
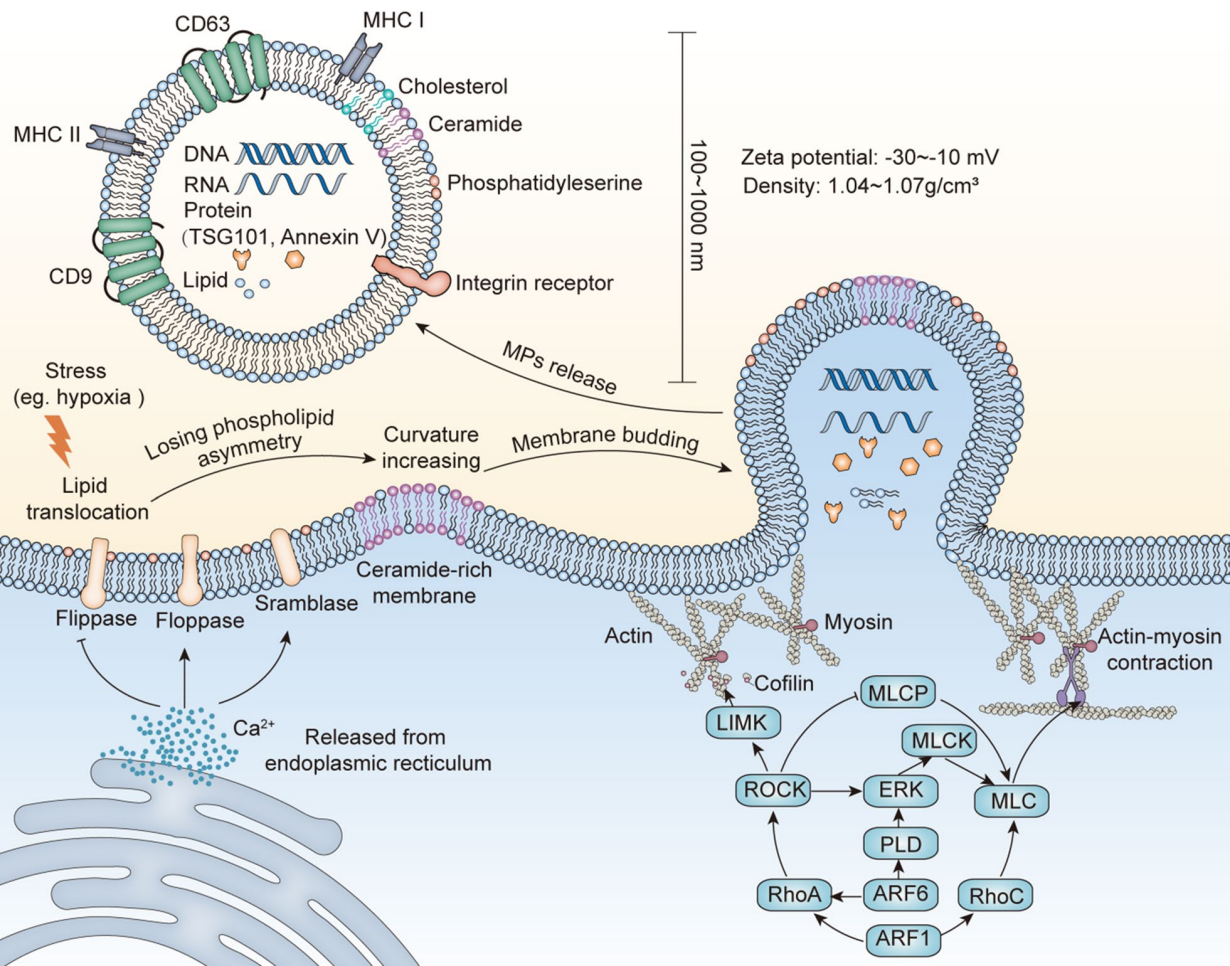
Biosynthesis and physiological functions of microparticles (MPs). Ca2^+^, released from the endoplasmic reticulum, regulates the activity of flippase, floppases and scramblases to translocate phosphatidylserine to the outer cell surface, modulating the asymmetry of membrane lipids and increasing curvature of the local membrane. Followed by actomyosin contraction, the outward budding of the membrane splits and MPs are released from the cell surface

To date, a detailed mechanistic understanding of MP formation is not available. We summarize the general process of MP biogenesis and describe it below. First, Ca^2+^ is released from the endoplasmic reticulum which activates several Ca^2+^-dependent enzymes, such as floppases and scramblases, to translocate phosphatidylserine to the cell surface [6]. As reported, except for phosphatidylserine exposure, transmembrane protein clustering and changes in lipid composition can also lead to the asymmetry of membrane lipids, which can further increase curvature of the local membrane [7, 8]. Subsequently, followed by actomyosin contraction, the outward budding of the membrane splits and MPs are released from the cell surface (Fig. 1). The actin-myosin-based contraction depends on adenosine triphosphate (ATP) and occurs at the neck of MPs. Therefore, the apical membrane slides toward the tip of the microvilli [9–11].

### Factors influencing MP biogenesis

Numerous studies have shown that the cytoskeleton plays an important role in various cellular biological activities, including the release and uptake of MPs [12–14]. Small GTPases, critical cytoskeletal regulators in cells, are important in actin activation (Fig. 1). ADP-ribosylation factor (ARF), a small G protein subfamily member, consists of six family members, ARF1-6. ARF1 activates both RhoA and RhoC, which phosphorylate myosin light-chain (MLC) and promote actomyosin contraction. Furthermore, RhoA activates RHO-associated protein kinase (ROCK), followed by the phosphorylation of Lim kinase (LIMK), which activates cofilin and prevents actin cleavage [15]. In addition, ROCK also activates extracellular signal-regulated kinase (ERK) and inactivates myosin light chain phosphatase (MLCP), thus maintaining the phosphorylation status of MLC [16]. In addition to ARF1, ARF6 is also capable of activating RhoA and promoting the activation of its downstream signaling pathways [17]. Phospholipase D (PLD), another downstream molecule of ARF6, not only directly phosphorylates MLC but also recruits ERK to activate MLC kinase (MLCK), resulting in the release of MPs [11]. Alternatively, ERK-dependent phosphorylation of chromosome segregation 1-like induces v-H-Ras expression and enhances MP biogenesis [18]. Moreover, peptidylarginine deiminase influences cytoskeletal rearrangement by mediating actin deimination [19, 20].

In contrast to the promoting effect, proteins or some small-molecule compounds that regulate the cellular cytoskeleton inhibit the synthesis and release of MPs. For instance, cytochalasin D inhibits F-actin polymerization, thus suppressing actin filament formation [21, 22]. Similarly, blebbistatin restrains actin filament motility, also resulting in decreased MP release [23]. In addition, Diaphanous-related formin-3, another cytoskeletal regulator, adjusts and controls the activation of cofilin, leading to the inhibition of membrane budding and MP release [24].

Furthermore, in addition to cytoskeletal regulators, some cell membrane receptors are also involved in the process of MP biogenesis and secretion. For example, activation of the purinergic receptor P2X7 by ATP facilitates MP release through the p38 mitogen-activated protein kinase (MAPK) and nuclear factor kappa-B (NF-κB) pathways, with acidic sphingomyelinase evagination and ceramide formation [25–27]. Additionally, as a protease-activated receptor (PAR), PAR2 mediates four distinct downstream pathways (P38/MK2/HSP27, RhoA/ROCK, MAPK/MLCK/MLC and AKT/Rab5a) to promote MP secretion [28, 29]. Other receptors, such as α-2-macroglobulin receptor [30], tissue factor [31, 32], G protein-coupled receptor 30 [33] and transient receptor potential vanilloid type 1 [34], also participate in the process of MP biogenesis and secretion.

Other factors that determine MP biogenesis include environmental or biochemical stimuli, such as changes in oxygen content or exposure to cytokines, which may activate various signaling pathways to elicit MP biogenesis. For instance, hypoxia increases the expression of the small GTPase Rab22a through hypoxia-inducible factors and mediates MPs formation, while tumor cells with normal oxygen levels do not upregulate hypoxia-inducible factors expression and still release MPs [35]. Tumor necrosis factor-α (TNF-α) is a common inducer for MP biogenesis in endothelial cells through TNFR1/NF-κB pathway [36].

## Properties of MP membranes

The net surface charge, as detected using the dynamic light scattering technique and characterized by zeta potential, is an important factor that affects the stability and half-life of MPs in blood circulation in vivo. Studies have shown that the surface charge of MPs is negative, and the specific value varies according to cell sources and disposal methods, normally between −30 and −10 mV and usually similar to the parental cells [37, 38]. The negative charge of MPs is mainly attributed to negatively charged lipids, especially phosphatidylserine [39]. In addition to prolonging half-life in vivo, multiple reports have shown that negative charges on MP surfaces also help enhance their blood compatibility and decrease their clearance by the reticuloendothelial system [40, 41]. Therefore, when modifying MPs, this feature should be maintained so that surface charge maintains a negative character.

**Fig. 2:**
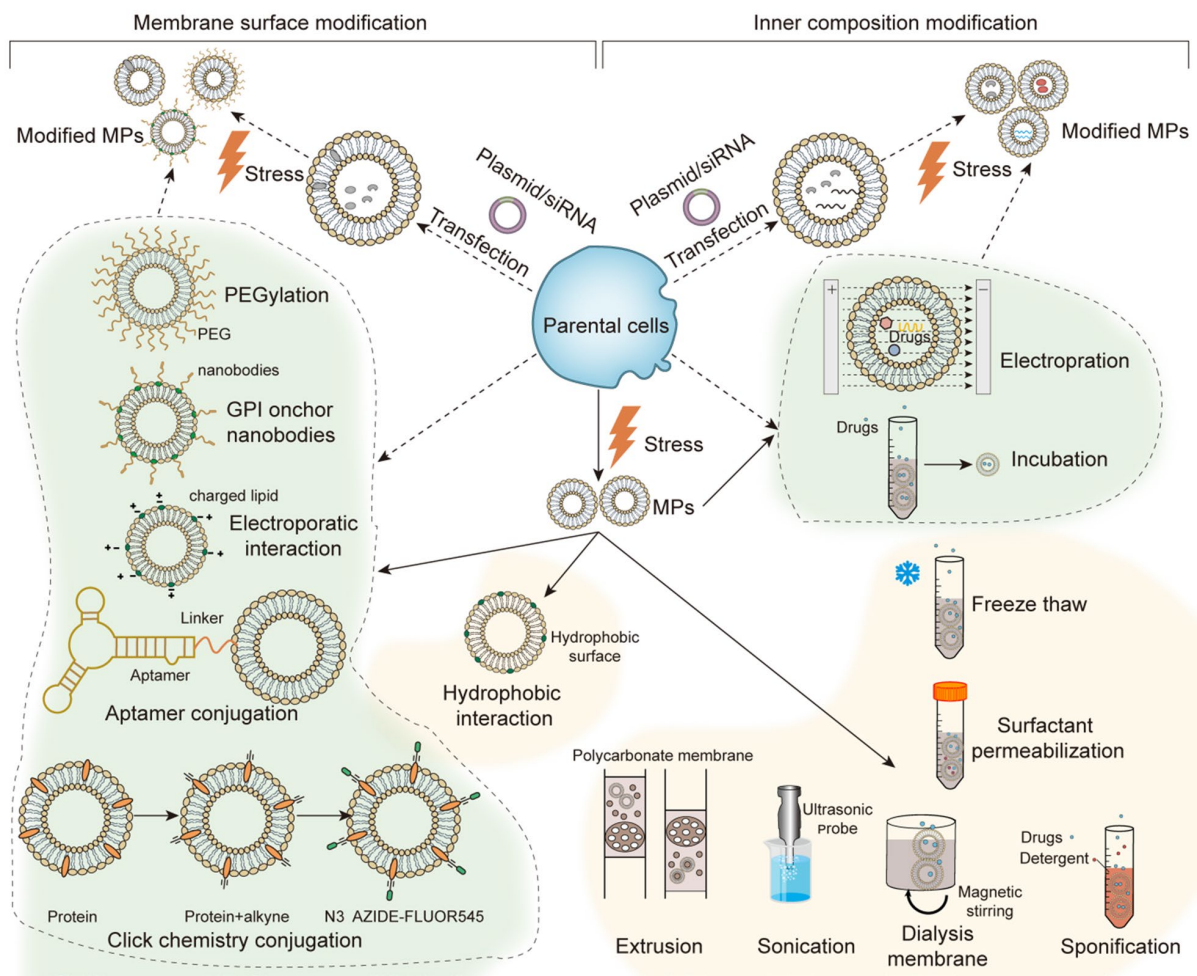
Modification strategies for the surface membrane and internal composition of MPs. Both the membrane surface (left) and inner composition (right) of MPs can be changed to enhance the function of MPs. The modification strategy can be separated into two categories. One is to modify the original cells, whose MPs have the same modification (dotted arrow). Another one is to modify MPs directly (solid arrows). Furthermore, the usage of modification methods is restrictive. Transfection can only be applied to cells. Methods with yellow background can only be applied to MPs. Methods with green background can be applied to both cells and MPs

With respect to biogenesis of MPs, the membrane molecules on MPs are similar to their parental cells, providing specific molecules as biomarkers for MP identification, such as externalized phosphatidyl serine, CD9, ARF6, tumor susceptibility gene 101 protein (TSG101) and CD63 [42–45]. However, these biomarkers are unable to distinguish EVs of different subtypes and further research is needed in this area. In order to enhance the biological function of MPs, various membrane surface modification for MPs have been investigated (Fig. 2). These modification methods can be divided into two categories. One is to modify the cell membrane of original cells and ensure that the secreted MPs have the same modification. The other one is to directly use the bilayer membrane structure of isolated MPs for modification. Details of these modifications are illustrated below in the intervention therapy section.

## Size and internal composition of MPs

As stated above, the diameter of MPs ranges from 100 to 1000 nm, which is much larger than EXOs (30–100 nm). A larger internal volume indicates that more substances from donor cells can be transported, potentially resulting in a stronger biological response. Larger payloads can be carried when MPs serve as vectors for drug delivery. Moreover, the appropriate size of MPs contributes to their passive tumor-targeting activities. This activity might be explained by the enhanced permeability and retention (EPR) effect, which is based on the difference between normal (5–8 nm) and tumor (100–780 nm) capillary gaps [46–48]. Unlike normal tissues, endothelial cells of the tumor vasculature are not tightly arranged, leading to high permeability of tumor blood vessels and facilitating the entrance of MPs into cancer sites. Furthermore, lymphatic drainage within tumors is insufficient, contributing to the passive accumulation of MPs in tumors.

In addition to membrane lipids and proteins, MPs usually package many biological components present in the cytosol and nucleus of original cells [49, 50]. Among all of the characterized contents, proteins [51, 52] and RNA [53] are the most well studied. The internal composition depends on a wide range of factors, such as the cell source and stimulation conditions [54]. As shown in Table 2, MPs derived from endothelial cells with starvation or TNF-α pre-treatment exhibited opposite functions in regulating expression of intercellular adhesion molecule 1 (ICAM-1), which suggested the high heterogeneity of MPs and more studies should be conducted in this area. Furthermore, it also indicated that components of MPs can be changed by performing modifications in the parental cells (Fig. 2). The common modification methods include incubation, electroporation and so on. Another way to alter the internal composition of MPs is directly packaging cargos into MPs through various methods, such as by physically or chemically breaking and reassembling the membrane of MPs (extrusion, freeze-thaw, etc.).

**Table 2:** Biological functions of different MPs

Original cells	Stress	Biological function	Refs.
Ordinary tumor cells	UV	Mediating M2 polarization of TAMs and promoting tumor growth and metastasis	[163]
		Loading PD-L1 and inhibiting activation of CD8^+^ T cells	[68]
		Facilitating the generation of type I IFN in DCs and promoting their activation through cGAS/STING signaling	[71]
		Up-regulating expression of CCL2 in IECs to attract and activate DCs for tumor inhibition	[59]
		Augmenting biogenesis and centripetal movement of lysosomes in tumor cells	[77]
	X ray	Causing ferroptosis in tumor cells and mediating M1 polarization of TAMs	[58]
		Activating stromal cells to secrete pro-angiopoietic factors	[66]
		Inhibiting antitumor immunity with the carried PD-L1	[67, 68]
	Hypoxia	Mediating M2 polarization of TAMs	[163]
		Up-regulating expression of CCL2 in lung macrophages and promoting lung metastasis	[164]
		Activating fibroblasts and endothelial cells to express pro-angiopoietic factors	[66]
		Inhibiting the functions of NK cells by transferring miR-23a	[165]
		Promoting focal adhesion formation, tumor invasion and metastasis	[35]
	No treatment	Inhibiting the activation of B cells and promoting the release of anti-inflammatory cytokines from monocytes	[166]
		Promoting antiapoptotic effect on monocytes by transferring CCR6 and CD44v7/8	[167]
		Promoting the differentiation of myeloid cells and enhancing their function on inhibiting T cells activation	[168]
		Inducing lymphocyte apoptosis by the carried FasL	[169]
		Promoting Treg differentiation and enhancing their negative regulation of immunity	[170]
		Promoting differentiation of monocytes to macrophages	[171]
		Up-regulating VEGF expression in endothelial cells by the carried epidermal growth factor receptor	[172]
		Inducing IL-10 production in monocytes by the carried hyaluronan	[173]
		Modulating antigen cross-processing in DCs through the packaged ROS	[174]
		Inducing premetastatic cell clusters and promoting liver metastasis by the carried CD36	[175]
		Converting normal fibroblasts into carcinoma-associated fibroblasts (CAFs) by the carried miR-155	[176]
		Up-regulating expression of transforming growth factor β in macrophages by the carried phosphatidylserine	[177]
	Starvation	Converting normal fibroblasts into CAFs by phosphorylating ERK1/2 and down-regulating caveolin1	[178]
		Up-regulating activity of focal adhesion kinase in epithelial cells and then reorganizing extracellular matrix	[179]
	Apoptogenic reagents	Activating fibroblasts through phosphorylating ERK1/2 and up-regulating MMP9	[180]
Chemo-resistant tumor cells	No treatment	Transferring resistance proteins to drug-sensitive tumor cells	[181–183]
		Down-regulating miR-503 and up-regulating proline-rich tyrosine kinase 2 of tumor cells to promote tumor migration and invasion	[184]
		Increasing the release of IL-6, TNF-α and INF-γ in macrophages	[185]
Stem-cell-like cancer cells	No treatment	Converting normal endothelial cells into an activated angiogenic phenotype and promoting the formation lung premetastatic niche	[186]
		Transferring tissue factor and accelerating plasma coagulation	[187]
Endothelial cells	Starvation	Activating angiogenesis in recipient cells by transferring mRNA	[188]
		Promoting anti-inflammatory effects by transferring miR-222 and reducing ICAM-1 expression in endothelial cells	[189]
	TNF-α	Up-regulating the expression of ICAM-1 in endothelial cells	[190]
		Converting endothelial cells into an anti-atherogenic phenotype by transferring miR-126, miR-21 and miR-155	[191]
		Inducing plasmacytoid DCs maturation and production of inflammatory cytokines Promoting proliferation and production of IFN-γ and TNF-α in CD4^+^ T cells	[192]
		Increasing monocyte adhesion by up-regulating the expression of ICAM-1 in endothelial cells Mediating apoptosis and inflammation of endothelial cells	[36]
CAFs	No treatment	Promoting generation of stem-cell-like cancer cells and resistance to hormonal therapy by transferring miR-221	[193]
Platelets	No treatment	Inducing angiogenesis through VEGF, heparanase, and platelet derived growth factor	[194]
		Stimulating proliferation and invasion of tumor cells by transferring integrin CD41	[195]
		Inducing epithelial to mesenchymal transition in tumor cells by transferring miR-939	[60]
		Mediating mitochondrial dysfunction and growth inhibition in tumor cells by transferring miR-24	[196]
		Promoting angiogenesis, tissue regeneration and cancer metastasis	[61]
	ADP	Increasing the production of lipoxin A4 in mast cells by transferring ipoxygenase 12	[197]
	High-shear	Increasing the expression of IL-8, IL-1β and TNF-α in macrophages Up-regulating the production of IL-8, IL-1β and IL-6 in endothelial cells	[198]
	TNF-α	Incapable of inducing plasmacytoid DCs maturation	[192]
Erythrocytes	Hypotonic solutions	Falsely “mark” nucleated cells as apoptotic by transferring phosphatidylserine	[42]
Monocytes	LPS	P-selectin glycoprotein ligand-1 on the MPs interacted with P-selectin on the platelets and activated platelets to initiate coagulation	[199]
		Promoting pro-inflammatory and procoagulant properties of endothelial cells by transferring transcripts of pro-inflammatory cytokines such as TNF-α, IL-6 and IL-8	[200]
	No treatment	Promoting angiogenesis by transferring miR-150 to endothelial cells	[201]
Macrophages	LPS	Inducing expression of ICAM-1 and release of keratinocyte-derived cytokine by transferring TNF-α	[202]
	Lm infection	Transferring antigens of Listeria monocytogenes (Lm) to DCs for antigen presentation	[203]
T lymphocytes	Inducers of apoptosis	Inducing apoptosis and stimulating release of MPs in macrophages	[204]
	Starvation	Converting fibroblasts into osteoclasts by up-regulating IL-15, MMP9 and receptor activator of NF-κB ligand in odontogenic keratocysts	[205]
	TNF-α	Incapable of inducing plasmacytoid DCs maturation	[192]
	UV	Incapable of mediating M2 polarization of TAMs	[163]
	No treatment	Inhibiting growth and migration of retinal endothelial cells in vitro, and decreasing VEGF-induced retinal vascular leakage in vivo	[206]
Splenic cells	PMA	Increasing tumor metastasis by transferring integrin α(M)β_2_	[207]
Yeast cells	NaOH and heat	Activating DCs through Dectin-1/Syk pathway and TLR2/MyD88 pathway	[78]

## Isolation methods of MPs

MPs have been isolated using a variety of methods, such as centrifugation, size exclusion chromatography, ultrafiltration, immunoaffinity chromatography, and microfluidics [55] (Table 3). All of these techniques can be used for isolation of both MPs and EXOs. Immunoaffinity chromatography is not able to distinguish these two types of EVs because exclusive markers have not yet been identified between them. Other methods that can separate EXOs and MPs include differential centrifugation (using centrifugation) or size exclusion chromatography, ultrafiltration or microfluidics (using variable pore or sieving size). Furthermore, some of these methods may be combined. For example, size exclusion chromatography can be followed by centrifugation or ultrafiltration to concentrate isolated but diluted MPs. Immunoaffinity chromatography often can serve as a further purification method after MPs isolation from large sample volumes. In clinical settings, the choice of separation method usually depends on the clinical purpose. For disease diagnosis, microfluidics is an appealing approach as it is fast, simple to operate, and available for small volume samples [56]. As for large scale production, ultrafiltration is more suitable due to the low cost and simple operation [57]. However, there are challenges in clinical grade utilization. The greatest is that there are no standardized protocols for MP isolation, purification, quantification and storage. Therefore, identification of specific markers to distinguish EVs of different subtypes will be beneficial for establishment of more accurate and effective isolation and purification approaches. For clinical testing, it is essential to standardize specimen source, handling method, and test volume according to test purpose. As for large scale manufacturing, the culture and storage conditions should also be standardized to ensure the reproducibility and stability of MPs, includes the culture medium components, solvents and buffers, storage bottles, storage temperature and shelf life.

**Table 3:** Comparisons of isolation methods

Method	Mechanism	Pros	Cons
Centrifugation	Stepwise centrifugation: removing cells and debris at a Low centrifugation speed (300–1000 g), followed by MPs collection at a higher one (10000–20000 g)	The most common and efficient isolation Method Low cost Simple to operate Not easily contaminated Wide range of sample volumes from a few millilitres up to > 100 mL	Low selectivity Risk of aggregation and deformation of MPs Risk of cosedimentation of larger vesicles and protein aggregates
Size exclusion chromatography	By using a column packed with porous gels, large EVs flow out first than small EVs. Each component is separated according to size	Reducing aggregation of MPs and proteins Maintaining integrity and biological activity of MPs Relatively high purification and inexpensive	In most cases, samples are diluted and re-concentration are required, resulting in long times If there are multiple production cycles, columns need to be washed, sanitized and rebalanced
Ultrafiltration	Use membranes with specific aperture to remove other components from the sample and retain and concentrate the MPs	Low cost Simple to operate Easy for large scale	Low selectivity Risks of non-specific binding of MPs to membranes and leading to some loss of yield Risks of deformation or rupture of MPs
Immunoaffinity chromatography	MPs are separated by the specific interaction of antigens on the surface of MPs and antibodies On the beads	High purification Capacity of isolating and quantifying specific sub-population of MPs	High cost Unsuitable for large-volume samples and uneasy for large scale Risks of deformation while elution Accurately sorting a particular type of MPs, thus losing the heterogeneity of MPs
Microfluidics	The isolation can be based on several aspects, such as shape, size, density, electric charge, specific lipid/proteins on MPs membrane	High purification Fast and simple to operate Capacity of isolating and quantifying specific sub-population of MPs in real time Available for small volume samples	Accurately sorting a particular type of MPs, thus losing the heterogeneity of MPs Risks of deformation on account of the shear stress

## Applications of MPs in cancer treatment

The lipid bilayer of MPs preserves the activity of entrapped contents, making these vesicles well-suited for antitumor therapy. To date, the application of MPs in cancer treatment can be divided into two categories: MPs as therapeutic agents themselves, and MPs as drug carriers for antitumor agents.

### MPs as therapeutic agents

As shown in Table 2, MPs derived from different cells or the same cells that had received different stimulations exhibited various biological functions. In general, MPs derived from tumor cells (T-MPs) suppress antitumor immunity, and promote angiogenesis and tumor growth and metastasis. Therefore, in most studies, T-MPs have been used as drug carriers. The exceptions are MPs derived from X-rays treated tumor cells (RT-MPs) [58] and MPs derived from ultraviolet-treated tumor cells (UT-MPs) [59], which are reviewed in detail further below. MPs that originate from platelets, which do not contain tumor antigens, are not expected to delay tumor progression themselves [60, 61]. Likewise, MPs derived from T lymphocytes were reported to increase tumor metastasis by transferring integrin α(M)β_2_ [62]. Although some types of MPs can activate the immune system (such as MPs derived from TNF-α-stimulated endothelial cells), their exact roles within tumors remains unknown. To date, MPs derived from non-tumor cells were seldom used for antitumor therapy alone, but often worked as drug carriers (Table 4). Therefore, more studies are needed to understand the exact biological effect of MPs derived from non-tumor cells to make better use of them for cancer therapy. Additionally, MPs from microorganisms stimulate immune responses due to their compositions that were recognized as pathogen-associated molecular patterns (PAMPs).

**Table 4:** Summary of the application of modified MPs in cancer therapy

Modified MPs	Original cells	Therapeutic agent and incorporation method	Metallic materials and incorporation method	Stress	Isolation method	Cancer type	Image	Refs.
Chemo@UT-MPs	Tumor cell	MTX /Cisplatin/DOX (incubation)	/	UV	Centrifugation	HCC, melanoma ovarian cancer, lung cancer, colon cancer	/	[84, 85, 130]
YM-155@ DOX@UT-MPs	Tumor cell	DOX and YM-155 (incubation)	/	UV	Centrifugation	Osteosarcoma	/	[134]
CCION/m-THPC@M-MPs	Macrophage	m-THPC (incubation)	Citrate-coated iron oxide nanoparticles (incubation)	Starvation	Magnetic sorting	Cervical cancer	MRI	[145]
QDs@VEGF siRNA@E-MPs	Endothelial cell	VEGF-siRNA (electroporation)	DSPE-PEG-biotin SA-QDs (incubation)	Starvation	Centrifugation	Melanoma	NIR	[142]
DOX@AS1411@E-MPs	Endothelial cell	DOX and AS1411-CHO (incubation)	/	Starvation	Centrifugation	HCC	/	[37]
Met@Man@UM-MPs	Macrophage	DSPE-PEG-Man and metformin (incubation)	/	UV	Centrifugation	HCC and breast cancer	/	[158]
OVs@UT-MPs	Tumor cells	Oncolytic adenovirus (infection)	/	UV	Centrifugation	Lung cancer, rectal cancer and ovarian cancer	/	[119]
Survivin siRNA@QDs@CMPs	Circulating MPs	Survivin siRNA (electroporation)	Ag_2_Se@Mn QDs/ electroporation	No treatment	Centrifugation	Oral cancer	MRI/NIR	[79]
DOX@FA/IONP@M-MPs	Macrophage	DOX (electroporation) and DSPE-PEG-FA (incubation)	DSPE-PEG-Biotin and SA-IONPs (incubation)	Starvation	Magnetic sorting	Cervical cancer	/	[153]
Bcl-2 siRNA/Taxol@FA/biotin@T-MPs	Tumor cell	Bcl-2 siRNA and Taxol (electroporation) DSPE-PEG-FA (incubation)	DSPE-PEG-Biotin and SA-QDs (CdSe/ZnS) (incubation)	Starvation	Centrifugation	Breast cancer	NIR	[155]
Chemo@UTT-MPs	TRCs	DOX or 5-FU (incubation)	/	UV	Centrifugation	HCC and melanoma	/	[38]
CpG@Fe_3_O_4_@UT-MPs	Tumor cell	CpG@Lipo (incubation)	Nano-Fe_3_O_4_ (incubation)	UV	Centrifugation	Melanoma	/	[148]
TK-NTR@T-MPs	Tumor cell	TK-NTR plasmid (transfection)	/	Starvation	Centrifugation	Breast cancer	/	[99]
Bi_2_Se_3_/DOX@UT-MPs	Tumor cell	DOX (electroporation)	Bi_2_Se_3_ (electroporation)	UV	Centrifugation	HCC	CT/PA	[138]

#### MPs from X-rays treated tumor cells

Radiotherapy (RT), a frontline treatment for approximately half of cancer patients, is not suitable for some diseases, such as malignant pleural effusion (MPE) and ascites, due to the high mobility of effusion, increasing the difficulty to formulate radiotherapy plans [63, 64]. Yang et al. irradiated tumor cells with a dose of 20 Gy by 6-MV X-rays and collected MPs from supernatants 72 h later (termed RT-MPs) to expand the clinical indications for radiotherapy [58]. They found that these RT-MPs contained more reactive oxygen species (ROS) than naturally secreted T-MPs and were able to kill tumor cells by causing ferroptosis. Intriguingly, the concentration of RT-MPs that killed cancer cells did not change the cellular activity of fibroblasts and even increased the proliferation of macrophages. Furthermore, intrapleural injections of RT-MPs significantly prolonged survival of MPE mice. Analysis of the tumor microenvironment (TME) showed that RT-MPs were mainly taken up by tumor-associated macrophages (TAMs) and repolarized them to the proinflammatory M1 phenotype, endowing them with a stronger capacity to phagocytose cancer cells. Nevertheless, programmed cell death ligand 1 (PD-L1), an important immune checkpoint that suppresses the activation of T cells and mediates immune tolerance toward malignant cells [65], was substantially upregulated in RT-MPs-treated TAMs, which prompted the combined treatment of RT-MPs and anti-PD-1. The combined treatment cured approximately 20% of cisplatin-resistant MPE mice without causing side effects, while cisplatin administration alone led to significant weight loss and abnormal changes in the levels of leukocytes, aspartate transaminase and alanine transaminase. This study confirmed that RT-MPs effectively expand the clinical indications for radiotherapy and provided evidence for the use of MPs as biological drugs. However, the limitation is that efficacy and security of RT-MPs in patients still remains to be tested in clinical trials. Since 20 Gy is a relatively high dose seldom actually applied in clinical settings, a lower radiation dose (10 Gy [66], 2 Gy [67], or 1 Gy [68]) has been used with tumor cells to produce MPs. In contrast to the 20 Gy-induced RT-MPs, a lower radiation dose induced RT-MPs was reported to exert a tumor-promoting effect through activating stromal cells to secrete pro-angiopoietic factors [66] and inhibiting antitumor immunity with the carried PD-L1 [67, 68]. These studies demonstrate that even if the stimulation is the same, different intensities may induce the formation of MPs with divergent functions.

#### MPs from UV-treated tumor cells

**Fig. 3:**
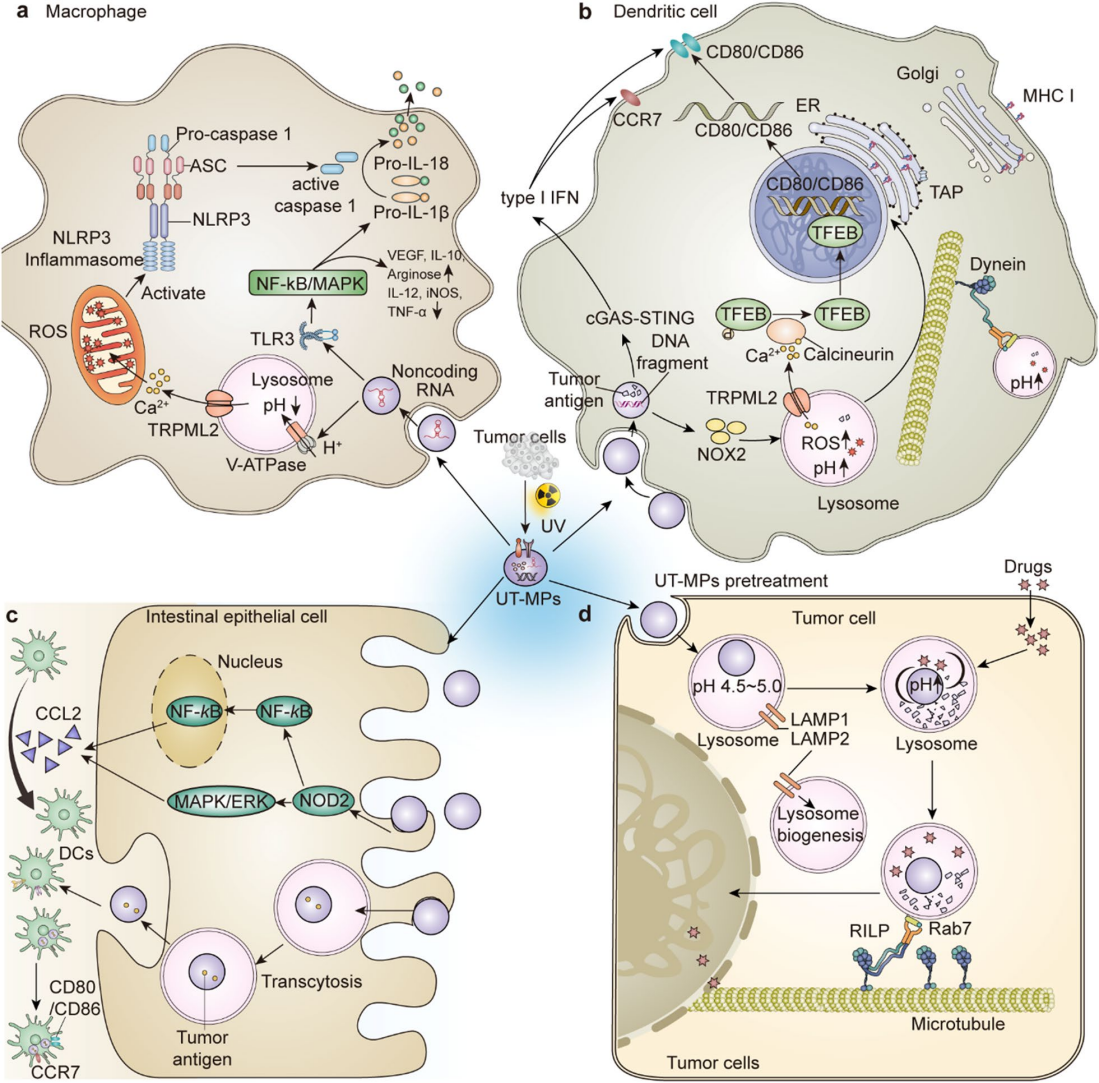
Biological functions of MPs from UV treated tumor cells (UT-MPs). **a**–**d** The biological effects of MPs after uptake by macrophages (**a**), DCs (**b**), intestinal epithelial cells (**c**) and tumor cells (**d**), respectively

Ultraviolet (UV) irradiation is one of the most common MP-inducing strategies used to date, and can elicit MP release. Huang et al*.* treated tumor cells with UV (300 J/m^2^) for 1 h and isolated MPs from supernatants 12 h later (termed UT-MPs). These UT-MPs contained an extensive repertoire of tumor-specific antigens, numerous DNA fragments and various types of RNA, all of which were ingested and exerted different biological effects on the target cells through different mechanisms (Fig. 3). For example, as professional phagocytes, macrophages were able to take up a mass of UT-MPs (Fig. 3a), after which the enriched noncoding RNAs activated TLR3/NF-κB and TLR3/MAPK pathways, promoting macrophages to express pro-IL-1β and polarization toward the M2 phenotype (with increased expression of CD206, interleukin-10 (IL-10) and arginase 1 and reduced expression of CD86, iNOS and TNF-α) [69]. UT-MPs endocytosis also increased the level of V-ATPase on the lysosomal membrane of macrophages, which pumped H^+^ from the cytoplasm to the lysosomal cavity [70] and decreased the lysosomal pH. Afterward, Ca^2+^ was transferred from the lysosomal cavity to the cytoplasm by mucolipin 2 (TRPML2), which upregulated ROS production in mitochondria and subsequently activated the NOD-like receptor family, pyrin domain containing 3 (NLRP3) inflammasome, enabling it to cleave and activate caspase1. Next, activated caspase1 cleaved the upregulated pro-IL-1β and finally enabled macrophages to secrete large amounts of IL-1β.

In addition to macrophages, dendritic cells (DCs), classic antigen-presenting cells, also take up UT-MPs (Fig. 3b). DNA fragments in UT-MPs facilitate the generation of type I interferon (IFN) in DCs through the cyclic GMP-AMP synthase (cGAS)/stimulator of interferon genes (STING) signaling pathway [71]. Type I IFN, in turn, promotes DC activation and returns to draining lymph nodes with the upregulation of costimulatory molecules and activation markers, such as CD80, CD86 and CCR7. Subsequently, the activated DCs presented tumor antigens to T cells and stimulated their proliferation, eliciting antitumor immunity. A detailed molecular mechanism of tumor antigen presentation and T cell activation was reported previously. Huang et al*.* observed that UT-MPs loaded in DCs increased the expression of NADPH oxidase 2 (NOX2) and recruited it to the lysosomal membrane to form a heterodimer with p22phox [72]. Then, NOX2-p22phox assembled into an active oxidase complex with cytosolic regulatory proteins, including GTP-binding Rac, p67phox, p40phox and p47phox, which transferred electrons from NADPH to dioxygen and subsequently generated superoxide anions [73]. The superoxide anion reacted with H^+^ and was reduced to hydrogen peroxide, leading to H^+^ consumption and an increase in the pH of lysosomes. Although the elevated lysosomal pH attenuated enzymatic activity and led to the generation of long peptides, which were unable to interact with major histocompatibility complex (MHC) class I for antigen presentation, UT-MPs increased the expression of both antigen peptide transporter 1 (TAP1) and TAP2, thus conveying MHC class I into the ER and promoting tumor antigen presentation. Moreover, followed by pH elevation, the small GTPase Rab7 and motor protein dynein were recruited to lysosomes, making lysosomes move centripetally and facilitated antigen cross-presentation [74]. Concurrently, UT-MPs endocytosed in DCs also increased the level of the Ca2^+^ channel TRPML2, triggering Ca2^+^ release from lysosomes to the cytosol. Therefore, calcineurin was activated and dephosphorylated the phosphorylated transcription factor EB (TFEB) [75], which translocated from the cytosol to the nucleus and bound to the promoters of CD80 and CD86, inducing CD80 and CD86 expression. Collectively, the antigen cross-presentation function of UT-MPs-treated DCs was significantly improved, resulting in more MHC-cancer antigenic peptide complex presentation and higher expression levels of costimulatory molecules on the cell surface, thus effectively activating T cells and eliciting specific antitumor immunity.

The studies described above suggest the application of UT-MPs for cell-free vaccines. As Huang et al. reported, subcutaneous vaccinations with UT-MPs triggered specific prophylactic protective effects from challenge with a wide variety of malignant cells, including H22 murine hepatocarcinoma tumor cells, CT26 colon carcinoma cells and B16 melanoma cells [71]. Interestingly, H22-UT-MPs inhibited the tumor growth of Hepa1-6 cells, another type of mice hepatocarcinoma cell line, but not CT26 cells, indicating that some similar tumor antigens in UT-MPs from the same type of malignant cells were able to be cross-presented by antigen-presenting cells. This feature may help simplify UT-MPs-based vaccination therapy in clinical applications. In addition, although tumor cell lysates and EXOs also contain large amounts of tumor-associated antigens [76], UT-MPs were more efficacious for vaccination in immunized mice. While subcutaneous delivery of UT-MPs fails to subdue the preexisting tumors, using DCs as a carrier for UT-MPs potentially reaches therapeutic efficacy, with greater frequency of CD8^+^ T cells infiltrating the tumor.

In addition to subcutaneous or intravenous injection of UT-MPs as a vaccine administration route, oral administration of UT-MPs has been shown to exert prophylactic and therapeutic effects on tumors [59]. The majority of oral UT-MPs are phagocytosed by ileac intestinal epithelial cells (IECs) (Fig. 3c), which are characterized by a high rate of E-cadherin positivity. After IECs take up UT-MPs, nucleotidebinding oligomerization domain 2 (NOD2) and its downstream signaling pathways are activated, inducing the incremental release of CCL2 and attracting CD103^+^CD11c^+^ DCs to migrate to the intestinal subepithelium, where IECs transferred UT-MPs to DCs through transcytosis. After loading tumor antigens in UT-MPs, DCs increase the proportion of IFNγ-excreting CD8^+^ and CD4^+^ T cells, inducing specific immune responses to suppress tumor growth.

UT-MPs were also shown to sensitize mice to intravesical chemotherapy through preinstillation in nonmuscle-invasive bladder cancer (NMIBC), without any abnormal changes in mouse hair, weight, liver or kidney functions [77]. The sensitizing effect of the UT-MPs pretreatment was not mainly due to immune activation, but to the increasing accumulation of drugs in the nucleus through a lysosomal pathway. UT-MPs preinstillation augmented lysosome biogenesis by upregulating the expression of lysosomal associated membrane protein 1 (LAMP1) and LAMP2 and increasing lysosomal pH through negative effects on vacuolar type H^+^ ATPase. Subsequently, Rab7 was recruited to lysosomes and facilitated their interaction with microtubules, triggering the centripetal movement of lysosomes. While chemotherapeutic drugs were transported to lysosomes after cellular uptake, those lysosomes moving toward the nucleus successfully transported the therapeutic drug into this organelle. Since UT-MPs were phagocytosed by cancer cells in the bladder (Fig. 3d), this specific improvement in toxicity to tumor cells significantly abrogated hematuria and prolonged the survival of bladder cancer-bearing mice.

**Fig. 4:**
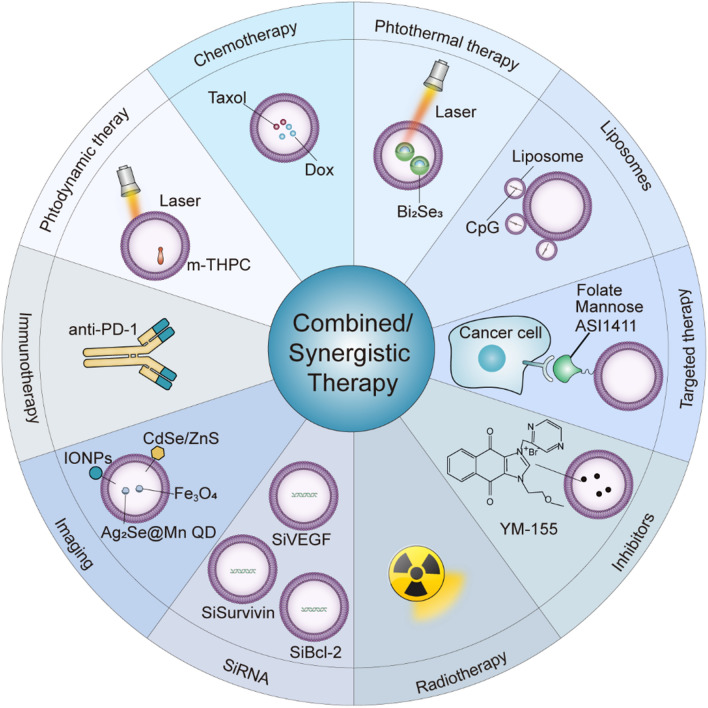
Combination or synergistic therapies mediated by MPs. Representative types of treatments of cancer are shown. MPs can mediate synergistic or combination treatment effects with these

Further research on the functional mechanisms of MPs is necessary. As displayed in Fig. 4, different cells will activate the same or different signaling pathways and generate different biological behaviors after the uptake of UT-MPs. Although UT-MPs increase the lysosomal pH of tumor cells and DCs, they also decrease the lysosomal pH of macrophages. The explanation for this discrepancy still remains to be explored.

#### MPs from yeast cells

Microorganisms contain some compositions that are considered as PAMPs by immune system. Wang et al. incubated yeast cells in NaOH at 80 °C for 1 h, followed by sonication and differential centrifugation to obtain EVs with different sizes [78]. These EVs contained ~ 88.2% β-glucan, 2.9% proteins and 8.9% other materials, and these activated DCs through Dectin-1/Syk and TLR2/MyD88 pathways. Furthermore, intratumoral injection of these yeast-cell derived EVs inhibited the growth of B16-luc tumor in vivo. Although MPs derived from yeast cells displayed inhibitory effects by intratumorally injection, the therapeutic effect of EXOs was better, on account of their small size, which facilitated their accumulation in tumor draining lymph nodes. This research lends support to the notion that microorganisms could be excellent parental cells for MPs preparation due to their immuno-stimulatary compositions. Also, the administration method was critical, and can directly determine the therapeutic effect.

### MPs as drug carriers

In recent years, several studies have reported the usage of EVs as DDS. Compared with artificial materials, EVs display certain advantages. First, EVs, as natural biomaterials, are less immunogenicity and less likely to induce side effects. Second, the biological molecules on the surface and inside EVs can induce biological effects, and rational selection will enhance the efficacy of delivered drugs. Third, in most cases, the solubility of drugs delivered by the artificial materials should be considered, while both water-soluble and hydrophobic drugs can be encapsulated into EVs through electroporation of cells, membrane fusion, or chemical conjugation. It should be noted that artificial materials also have their own advantages, such as the integration of therapy and bioimaging. Such benefits also can be conferred to EVs by inclusion of those artificial materials.

As a subtype of EVs, EXOs and MPs have distinct physicochemical and pharmacokinetic properties as drug delivery vehicles. First, under otherwise similar conditions, the size of MPs released by cells are much larger than EXOs, which makes MPs more readily available and allows more cargoes packaged inside. It was reported that compared with EXOs, fewer MPs were required to prepare the same concentrations of drugs [79]. Second, MPs and EXOs display different half-lives with intravenous injection. As reported, EXOs derived from HEK293T cells were almost completely cleared from blood 6 h after the tail vein injection [80]. However, MPs, collected from peripheral blood of cancer patients, could be detected in circulation for more than 48 h after intravenous injection in mice [79]. Third, MPs potentially feature better tumor-targeting capacity during systemic administration; 24 h after intravenously injection in mice, the accumulation of EXOs in tumors was less than 10% [81, 82], while MPs reached 19% [79]. In summary, compared with EXOs, MPs demonstrate some advantages for drug delivery, including greater loading capacity, longer half-lives in circulation and better tumor-targeting during systemic administration, however there has been less research regarding MPs compared to EXOs [83]. Therefore, MPs appear to be underutilized for drug delivery applications and warrant more investigations.

#### Single drug loading

*Chemotherapeutic drug loading* Chemotherapy, one of the conventional treatment approaches for advanced cancers, makes use of chemical substances to induce tumor cell death through different mechanisms, such as inducing DNA interstrand cross-links, free radical production, cell cycle arrest and double strand breaks. Chemotherapeutic drugs can be packaged into UT-MPs (chemo@UT-MPs) through incubation with tumor cells before UV irradiation, forming a delivery system for chemotherapy drugs, including methotrexate (MTX), doxorubicin (DOX), cisplatin, and hydroxyl camptothecin. The loading efficiency depends on several factors, such as the types and concentrations of chemotherapeutic drugs and donor cells. The higher the concentration, the more drugs the UT-MPs delivered. In addition, the size distribution of chemo@UT-MPs varied among different medicines, but the output was usually similar. Additionally, chemo@UT-MPs can be stored at 4 °C for 1 week and are stable and resistant to shaking, alkaline and acidic environments, room temperature, Triton X-100 and light, but not to proteinase K and sodium dodecyl sulfate [84].

**Fig. 5:**
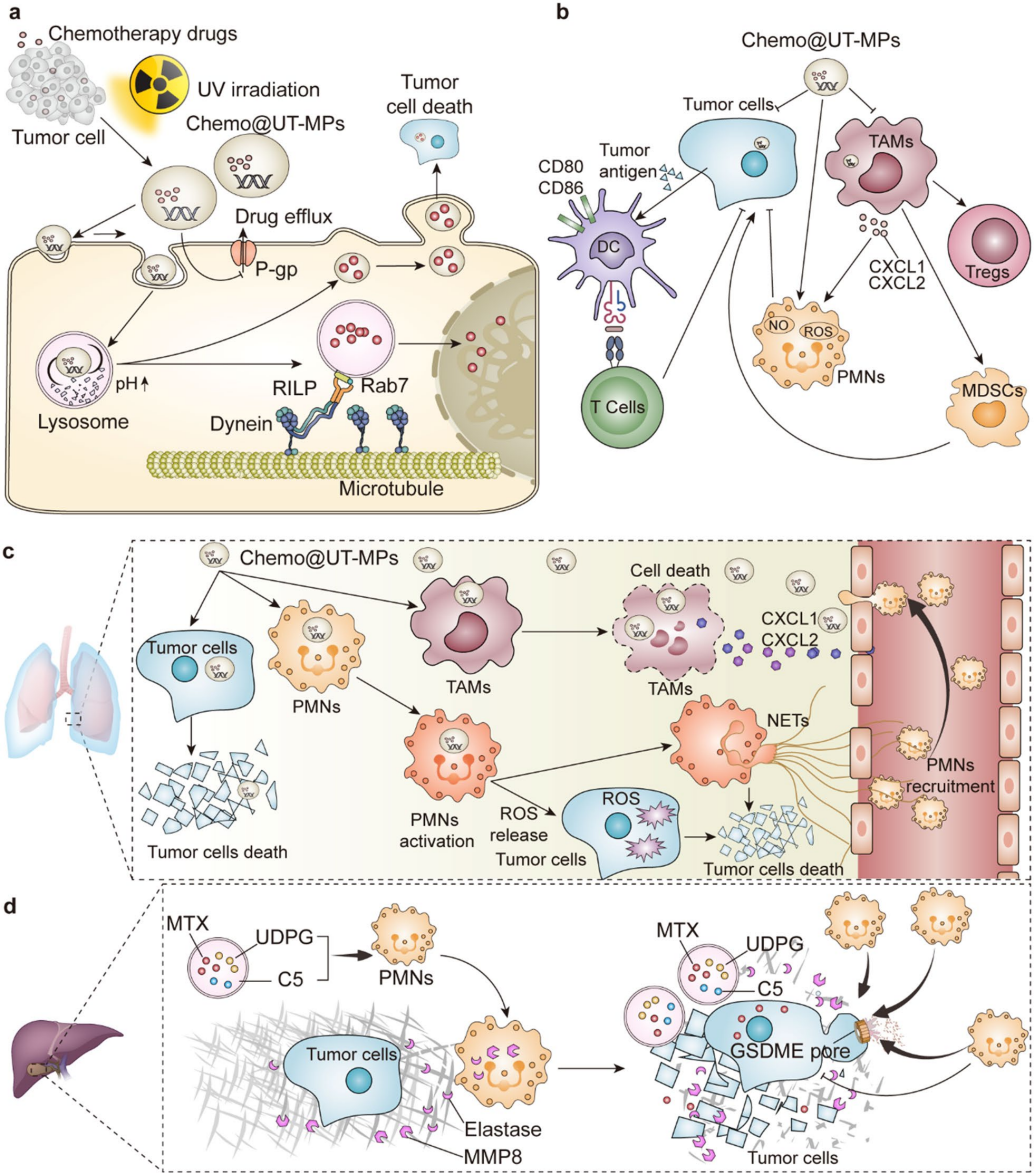
Biological functions of chemotherapeutic drug-loaded UT-MPs (chemo@UT-MPs). **a** The killing mechanism of chemo@UT-MPs on tumor cells. **b** Influence of chemo@UT-MPs on tumor immune microenvironment. **c** Chemo@UT-MPs in the treatment of malignant pleural effusion (MPE). **d** Chemo@UT-MPs in the treatment of cholangiocarcinoma (CCA) and extrahepatic malignant biliary tract obstruction

Although the drug-loading of chemo@UT-MPs seems to be low (0.52% and 2.5% for DOX@UT-MPs and MTX@UT-MPs, respectively), killing effect on tumor cells in vitro was superior to that of the free drugs at the same concentration [84]. For example, the number of tumor cells killed by DOX@UT-MPs was eight times higher than the number killed by the same dose of free DOX. Notably, in addition to killing ordinary tumor cells, chemo@UT-MPs even effectively kills drug-resistant stem cell-like tumor cells (also named tumor-repopulating cells, TRCs), which take up large amounts of chemo@UT-MPs on account of their softness and deformability [85]. Several key explanations for the strong killing efficiency of chemo@UT-MPs have been proposed (Fig. 5a). First, UT-MPs, inducing a mechanism to protect tumor cells from chemotherapeutic drugs, are actually concentrated and contain higher concentrations of drugs than tumor cells. Moreover, since many chemotherapeutic drugs interrupt nucleic acid metabolism and work in the nucleus, UT-MPs potentially facilitate their entrance into the nucleus with the involvement of microtubule network, thus allowing more drugs to accumulate in the nucleus for tumor killing. Second, tumor cells treated with chemo@UT-MPs further secrete newly formed chemo@UT-MPs, which still contain the initial drugs, and exert cytotoxic effects on other tumor cells, resulting in a domino-like cancer killing effect. Finally, after the integration of UT-MPs membranes into the tumor cell membrane, the expression of drug efflux pumps that mediate multidrug resistance, such as P-glycoprotein (P-gp), is downregulated, sequentially restraining drug efflux and enhancing drug retention.

The antitumor effect of chemo@UT-MPs in vivo has been verified in a variety of mouse models, including intraperitoneal injection for malignant ascites, intrapleural injection for MPE, and intravenous injection for subcutaneous solid tumors. Their therapeutic effect in vivo is not only mediated by the direct killing of tumor cells but also the activation of the immune system (Fig. 5b). In the TME, chemo@UT-MPs are mainly taken up by TAMs and tumor cells, resulting in their death. Considering the important role of TAMs in tumor development by promoting angiogenesis, remodeling the TME and damaging antitumor immunity [86, 87], the death of TAMs might be one of the mechanisms by which chemo@UT-MPs cure cancers, while the use of clodronate liposomes to deplete TAMs improves the antitumor effect of chemo@UT-MPs. Meanwhile, dead tumor cells activate DCs, increasing the expression of CD80 and CD86. Subsequently, chemo@UT-MPs increase the frequency of infiltrating T cells and neutrophils (PMNs), which contribute to transforming the immunosuppressive TME into an immunologically activated TME.

Based on these results, Huang et al*.* delivered chemotherapeutic medicines using TRC-derived MPs (chemo@UTT-MPs), which showed superior antitumor efficacy than chemo@UT-MPs that originated from differentiated tumor cells [38]. Consistent with the softness and deformability of TRCs, UTT-MPs also possessed these features with the involvement of cytospin-A, a regulator of actin cytoskeleton reorganization [88]. As a result, chemo@UTT-MPs, rather than chemo@UT-MPs, were more prone to be taken up by TRCs, leading to increased chemotherapeutic drug accumulation and a more efficacious antitumor effect. Apart from the cancer targeting ability, chemo@UTT-MPs were also less toxic to macrophages, which preferentially phagocytose rigid microparticles, such as stiffer UT-MPs. As expected, chemo@UTT-MPs easily extravasated from tumor vessels and accumulated and deeply penetrated solid tumors, triggering strong anticancer efficacy in vivo*.* Nevertheless, despite the increase in the delivery rate, the clinical application of chemo@UTT-MPs was still restricted due to the availability of abundant TRCs, which were located in the center and hypoxic site of solid tumors [89, 90].

Moreover, considering the species differences between humans and mice, the clinical therapeutic effect of chemo@UT-MPs is noteworthy. Currently, three clinical trials of chemo@UT-MPs in the treatment of MPE in lung cancer were published in 2016 [85], 2019 [91] and 2020 [92] (Table 5). In these three studies, 3, 11, and 32 patients with advanced lung cancer and MPE received chemo@UT-MPs treatment. The objective response rates reached 100%, 90.91% and 84.38%, respectively, indicating the excellent therapeutic effect of chemo@UT-MPs on humans. Commonly, the MPE volumes of some patients were reduced and MPE-related symptoms, such as gasp and cough, were highly relieved. The red color of MPE turned lighter, suggesting the presence of fewer erythrocytes and improved vascular permeability in MPE. More specifically, some differences in the production of chemo@UT-MPs were noted. For instance, drugs packaged in UT-MPs were cisplatin or MTX, and the original cells were the human lung carcinoma cell line A549 or autologous tumor cells collected from each patient’s MPE. Regarding the specific treatment plan, patients were treated with chemo@UT-MPs four or six times and regularly monitored with computerized tomography and ultrasound. In addition, the mechanism of chemo@UT-MPs in the treatment of MPE has been generally clarified (Fig. 5c). In addition to the direct killing and immune activation effects mentioned above, chemo@UT-MPs were also beneficial for damaged pleural capillaries by attracting PMNs to anchor to blood vessels and activating them to form neutrophil extracellular traps (NETs). The filamentous reticular structure and viscosity of NETs substantially contribute to the repair of leaky blood vessels in the thoracic cavity.

**Table 5:** Clinical trials of MPs-based cancer therapy

Disease	Drug	MPs source	Phase, *n* of patients	Objective response rate	Refs.
Malignant Pleural Effusion	MTX	Autologous tumor cells	Phase 2, *n* = 11	90.91%	NCT02657460 [91]
Malignant Pleural Effusion	Cisplatin	Tumor cells	Phase 2, *n* = 6	100%	NCT01854866 [85]
Malignant Pleural Effusion	MTX	Tumor cells	Phase 2, *n* = 32	84.38%	ChiCTR-ICR-15006304 [92]
Cholangiocarcinoma	MTX	Tumor cells	Phase 2, *n* = 20	100%	ChiCTR-OIB-15007589 [93]

In addition to MPE treatment, chemo@UT-MPs have also been applied in patients with cholangiocarcinoma (CCA) and extrahepatic malignant biliary tract obstruction [93]. In this study, 20 patients were treated with MTX@UT-MPs, which were injected into the bile duct cavity above the tumors through percutaneous transhepatic biliary drainage. Most patients responded to this treatment, and bile duct obstruction was relieved in 25% of patients. While investigating the underlying treatment mechanism (Fig. 5d), the direct killing effect of MTX@UT-MPs on tumor cells was not easily observed in the early stage due to the hard extracellular matrix of CCA. Only after the recruitment of PMNs to bile through nucleotide sugar uridine diphosphoglucose (UDPG) and C5 in UT-MPs were the stromal barriers degraded by elastase and matrix metalloproteinase 8 (MMP8). Hence, MTX@UT-MPs accessed malignant cells and destroyed them. Moreover, the death of tumor cells caused by MTX@UT-MPs was pyroptosis via the gasdermin E (GSDME) pathway, with large bubbles sprouting from the plasma membrane and large amounts of the cytosolic components released, such as lactate dehydrogenase [94, 95]. Subsequently, substances released during pyroptosis triggered a secondary wave of migrating PMNs, which were capable of killing tumor cells through elevated ROS and nitric oxide (NO) production, displaying an antitumor phenotype.

Although chemotherapy is limited by its side effects in clinical treatment, such as myelosuppression and alopecia [96, 97], chemo@UT-MPs treatment was reported to be safer. In various mouse models and in patients with advanced lung cancer and MPE, chemo@UT-MPs causes few side effects, such as any damage to liver and kidney functions. Approximately 70% of patients with CCA presenting extrahepatic malignant biliary tract obstruction had a transitory fever but no other symptoms of discomfort, such as abdominal pain, diarrhea or vomiting. The good biocompatibility of chemo@UT-MPs might be due to the membrane structures of UT-MPs, which were formed from the original cell membranes.

*Nucleic acid loading* Nucleic acids, including DNA and RNA, are some of the most important substances of life. Given the different structures and mechanisms of nucleic acid molecules, their delivery efficiency by MPs may also be different. Contag et al*.* transiently transfected siRNAs, plasmid DNA (pDNA) and mRNA into HEK293FT cells and collected MPs 2 days later to compare the delivery function of MPs, which were validated to contain these nucleic acids. However, after delivery into recipient cells, pDNA was the most functional in promoting protein expression in acceptor cells (the delivered siRNA did not silence the expression of the target gene) [98]. Based on this observation, Contag et al*.* further developed an efficient strategy for protein enrichment in MPs through genetic engineering. They transfected 4T1 cells with minicircle DNA that encodes a thymidine kinase (TK)/nitroreductase (NTR) fusion protein, which converts prodrugs into active cytotoxic agents [99]. They confirmed the amount of TK/NTR in MPs through fluorescence imaging. Following intravenous injection, the TK/NTR-packaged MPs and prodrugs effectively inhibited tumor growth in vivo. Besides pDNA, the functional mimics of some key virus proteins was also able to enrich desired proteins in MPs. Liu et al*.* transfected vesicular stomatitis virus G protein (VSV-G) into host cells, which increased the production of MPs more than 1000-fold [100]. On this basis, they further encapsulated large macromolecules into MPs by split GFP complementation and showed that this delivery strategy could avoid lysosomal degradation, which increased the delivery efficiency.

However, in another study, RNA was also shown to be functional when delivered by MPs. Shi et al*.* proved that miRNA mimics packaged in MPs regulate the expression of target proteins and alter the biological behavior of tumor cells in vitro [101]. In addition, tumor cells often overexpress some genes that enhance tumor proliferation, mediate tumor drug resistance, and enhance tumor angiogenesis. An siRNA can degrade the mRNA encoded by these genes, resulting in tumor growth suppression through various signaling pathways. Unfortunately, siRNAs are easily degraded, and their half-life in the body should be prolonged through some means, such as MPs delivery. Current studies usually combine siRNAs with other therapeutic modalities, which will be introduced in subsequent combination therapy.

In addition, oncolytic virotherapy, utilizing the propagation capacity of viruses to destroy tumor cells, is regarded as a highly promising treatment to cure cancers [102, 103]. To date, oncolytic herpes simplex virus has been approved to treat advanced melanoma by the Food and Drug Administration (FDA) [104, 105]. Nevertheless, some impediments still exist that restrain the therapeutic effect of oncolytic virotherapy, including limited access to tumor cells because of the small number of virus-recognizing receptors, rapid intracellular activation of antiviral mechanisms against entering viruses, fast virus removal by antiviral antibodies and an inability to treat multiple metastatic tumors [106–109]. Moreover, side effects, such as paralysis and encephalitis, caused by antiviral immune responses also restrict the clinical application of this therapeutic modality [110]. Consequently, an ameliorative delivery system for oncolytic viruses is needed to overcome these obstacles. Concurrently, man-made nanoparticles or cells, including myeloid-derived suppressor cells (MDSCs), mesenchymal stem cells (MSCs), irradiated tumor cells and cytokine-induced killer cells, have been utilized to deliver oncolytic viruses [48, 111–114]. Nevertheless, each approach has corresponding limitations, such as the immunological ejection caused by “nonself” components in man-made nanoparticles and the difficulty of lysing carrier cells to discharge oncolytic viruses at the tumor site [115].

Because EVs naturally contain viruses [116, 117] and mediate their infections [118], researchers have conjectured that MPs would be highly desirable to deliver oncolytic viruses. In 2016, Huang et al*.* developed a UT-MPs-based oncolytic virus (OVs@UT-MPs) delivery system [119]. By incubating oncolytic adenoviruses with tumor cells for 48 h, the cells became rounder and detached from the petri dish; at this time point, the supernatants were gathered for OVs@UT-MPs isolation. OVs@UT-MPs indeed loaded oncolytic adenoviruses, whose viral genes were still functional and expressed at high levels. Furthermore, OVs@UT-MPs efficiently taken up by tumor cells were able to productively replicate and induce stronger cell death even than free oncolytic viruses, both in immunodeficient and immunocomplete mouse models. The better therapeutic efficacy of OVs@UT-MPs was based on the capacity of UT-MPs to prevent the clearance of oncolytic viruses by antiviral antibodies. In immunodeficient mice, the reduced cytotoxicity of oncolytic adenovirus was due to the decreased expression of coxsackie-adenovirus receptor (CAR), which participated in inducing the entry of oncolytic adenovirus into cancer cells [120], while OVs@UT-MPs overcame this downregulation and effectively transmitted oncolytic adenoviruses into malignant cells through endocytosis or membrane fusion. Moreover, UT-MPs also promoted the accumulation of oncolytic adenoviruses in caryons, where the viruses replicated and assembled. In addition, while oncolytic adenoviruses were suggested to target cancer stem cells (CSCs) [121], OVs@UT-MPs were validated to kill CSCs even more efficiently. In terms of biosafety, no kidney or liver dysfunction was observed in OVs@UT-MPs-treated mice. Taken together, this OVs@UT-MPs delivery system with substantial advantages would be a promising clinical treatment to target malignancies.

*Loading of metallic materials* Due to their large specific surface area and porosity, metal–organic frameworks are often used as carriers of antitumor drugs to improve their targeting ability toward tumor tissues [122, 123]. At the same time, some metal materials have also been used for imaging, thus achieving the integration of cancer diagnosis and treatment [124]. However, the biocompatibility of these materials sometimes severely limits their applications. In 2010, Gazeau and colleagues observed that once taken up by macrophages or monocytes, iron-oxide maghemite nanoparticles encapsulating MPs are released upon stimulation and rapidly and simply collected through magnetic sorting, suggesting an MPs delivery method for metallic materials [125].

Cells labeled with magnetic nanoparticles enable noninvasive MR imaging and tracking of cell migration [126]. Gazeau et al*.* incubated anionic citrate-coated Fe_3_O_4_ nanoparticles (AMNPs) with endothelial cell-derived MPs (E-MPs) for 1 h [127]. Through nonspecific electrostatic interactions, AMNPs bound to the surface of E-MPs (forming AMN@E-MPs), which were isolated, investigated, and manipulated by magnetic methods and imaged by magnetic resonance imaging (MRI) in vitro. However, since AMNPs might mask membrane-associated proteins and thus influence the biological functions of AMNP/E-MPs, the authors altered the manufacturing method and incorporated AMNPs into E-MPs by incubating AMNPs with endothelial cells for 1 h following 24 h of starvation [128]. Thus, endothelial cell-derived and AMNP-containing MPs (AMNP@E-MPs) were collected via centrifugation, and their MR imaging function was confirmed through an intravenous injection into the tail vein of model mice. Upon these discoveries, in 2013, Amanda et al*.* encapsulated a series of nanoparticles into E-MPs to design a theranostic nanomaterial delivery system [129]. Human umbilical vascular endothelial cells (HUVECs) were incubated with various nanoparticles, including quantum dots (QDs), gold nanoparticles (AuNPs), iron oxide nanoparticles (IONPs), iron oxide nanocubes (IONCs) and gold/iron oxide heterodimers (Au/IONPs), alone or in combination, followed by starvation treatment to elicit MPs release and establish this system. After magnetic sorting (magnetic field gradient of 55 T m^−1^), the size of nanoparticle-encapsulated MPs was detected using TEM and dynamic light scattering analysis, and the magnetic targeting potential was examined in magnetophoresis experiments. The diameters and magnetophoretic mobilities of these magnetic MPs varied from nanomaterial to nanomaterial. Moreover, all of them exhibited extremely efficient MRI detection and great heating performances, generating sufficient heat that was reported to induce cell damage. However, this system did not exert a therapeutic effect in vivo, and further studies are needed.

### Mediating synergistic/combination treatment

#### Chemotherapeutic drugs in combination with other treatments

*With low-dose irradiation* As mentioned above (Fig. 5a), MPs enhance the cytotoxicity of chemotherapeutic drugs toward tumor cells in several ways. Based on these studies, Huang et al*.* established a combination therapy of chemo@UT-MPs and low-dose irradiation (2 Gy × 2, once per 3 days), which further facilitated drug retention in the nucleus of tumor cells and thus generated a stronger killing effect on TRCs [130]. Accomplished by the incremental attenuation of TRCs, fewer macrophages were polarized into the tumor-promoting phenotype, which triggered a more immunoreactive TME, where immunosuppressive MDSCs and Tregs were both suppressed and T cells were recruited and became active with the upregulation of IFN-γ. Correspondingly, the expression of RANTES, the major TH1 chemokine, was increased and the expression of the major TH2 chemokine was decreased. This study also provided evidence to support the use of concurrent chemoradiotherapy, the currently most common clinical treatment for many different cancers.

*With small-molecule inhibitors* In addition to low-dose irradiation, other methods have been developed to overcome the drug resistance of malignant cells. Drug-resistant cancer cells often upregulate the expression of some proteins [131], which promotes the use of a combination of chemotherapy with targeted small-molecule inhibitors. For example, survivin, a member of the anti-apoptotic protein family, was upregulated and involved in drug resistance in a variety of tumors [132, 133]. Liu et al*.* incubated tumor cells with both chemotherapeutic drugs and a survivin inhibitor, YM-155, followed by UV irradiation to increase the release of MPs [134]. After stepwise centrifugation, the UT-MPs that encapsulated both DOX and YM-155 (YM-155@ DOX@UT-MPs) were collected. This new approach was confirmed to reverse drug resistance, augment antitumor effects and decrease systemic toxicity in a subcutaneous tumor model of osteosarcoma. Using this delivery system, we can easily extrapolate that many inhibitors with other functions can also be used in combination with chemotherapeutic drugs to improve their antitumor activity through different mechanisms.

*With photothermal therapy* Photothermal therapy, by irradiating materials with high photothermal conversion efficiency to convert light energy into heat, has been extensively investigated for tumor treatment [135, 136]. According to a study by Cheng et al*.,* intravenous injection of gold nanostar (GNS)-loaded MSCs inhibits prostate tumor growth to some extent. However, the large size still restricts the entrance of MSCs into the TME. GNS-loaded MSCs release GNS-encapsulated MPs and further transport them to recipient cancer cells in vitro [137]. Based on these results, Yang and coworkers invented an MPs-based multifunctional DDS that not only synergistically combined chemotherapy and photothermal therapy but was also equipped with photoacoustic (PA) and computed tomography (CT) imaging capacity [138]. They prepacked Bi_2_Se_3_ nanodots and DOX into tumor cells through electroporation and subsequently irradiated them with UV light to induce the release of both Bi_2_Se_3_ and MPs encapsulating DOX (Bi_2_Se_3_/DOX@UT-MPs). By changing conventional incubation into electroporation at 300 V and 150 µF, both the drug-packaging efficiency and production yield of Bi_2_Se_3_/DOX@UT-MPs were significantly increased. In addition, through the membrane fusion effect of MPs, Bi_2_Se_3_/DOX@UT-MPs markedly target cancer cells, deeply penetrate into 3D tumor spheroids and exhibit enhanced photothermal performance. Consequently, Bi_2_Se_3_/DOX@UT-MPs plus near infrared ray (NIR) irradiation treatment display desirable tumor suppressing activity, both in vitro and in vivo. Because of the low dose of DOX, this new platform for dual-modal imaging-guided synergistic cancer therapy had few side effects.

*With targeted therapy* In addition to using the EPR effect of MPs, scientists have also developed other methods to increase MPs aggregation in tumor tissues. Many tumor cells overexpress some proteins on the cell membrane [139, 140], suggesting that targeting these proteins will increase the accumulation of MPs in the TME and ingestion in tumor cells. For example, the oligodeoxynucleotide aptamer AS1411 specifically recognizes and binds nucleolin, a protein that is often overexpressed on the tumor cell membrane [141]. Based on this discovery, Mao et al*.* capped the aptamer AS1411 with an aldehyde end group (forming AS1411-CHO) and incubated E-MPs with AS1411-CHO for 4 h at 37 °C [37]. The coupling reaction between - NH_2_ groups of membrane proteins on E-MPs and aldehyde groups covalently conjugated AS1411-CHO to E-MPs (forming AS1411@E-MPs). Fluorescent imaging experiments showed that the aptamer AS1411 effectively increased and prolonged E-MPs accumulation in the TME. To effectively kill tumor cells, DOX was packaged into AS1411@E-MPs through incubation, forming DOX@AS1411@E-MPs, which markedly destroyed subcutaneous liver cancer cells after an intravenous injection without causing any toxic reactions. In addition to nucleolin, many other proteins are overexpressed on tumor cell membranes. Screening one with the highest expression or simultaneously targeting multiple membrane proteins may be the direction of future research.

*siRNAs in combination with other treatments* RNA inference technology has shown promise in therapeutic applications since its discovery. Although scientists have proven that RNA packaged in MPs is still functional [101], the amount of RNA also substantially alters the biological effects. Zhao et al*.* directly incorporated a vascular endothelial growth factor (VEGF) siRNA into biotinylated E-MPs through electroporation at 250 V and 350 μF to increase the siRNA concentration in MPs [142]. After an incubation with streptavidin-QDs (SA-QDs), QD-labeled and VEGF siRNA-encapsulating E-MPs (QDs@VEGF siRNA@E-MPs) were formulated. As confirmed, QDs@VEGF siRNA@E-MPs markedly decreased levels of both the VEGF mRNA and protein and substantially increased cell apoptosis in vitro. In the A2058 human melanoma xenograft mouse model, QDs@VEGF siRNA@E-MPs exhibited an excellent imaging function and great cancer-inhibitory effects without causing weight loss when intratumorally injected. This work described a safe and efficient strategy for siRNA visualization and delivery.

The MPs mentioned above were released from cells cultured in vitro, which is time- and labor-consuming and might occur with cross-contamination. Due to these concerns, Chen et al*.* first proposed the utility of circulating MPs (CMPs), which are mainly secreted by the vascular endothelium and multiple blood cells, such as erythrocytes, monocytes and platelets [143]. As reported, CMPs concentrations are substantially increased in patients with malignant cancers, which not only meet the large-scale demand in clinical treatment but also avoid cell culture and pretreatment processes. The authors labeled CMPs with Ag_2_Se@Mn QDs (QDs@CMPs) through electroporation to achieve dual-mode traceability and to identify the biological functions and biodistribution of CMPs in vivo [79]. As observed, the photostability of QDs@CMPs was excellent both in MRI and NIR imaging. Compared with the Ag_2_Se@Mn QDs intravenous injection, QDs@CMPs exhibited a much longer circulation half-life and accumulated in tumors at a higher level in the CAL27 xenograft mouse model. Two potential explanations for this tumor-targeting capability have been proposed. First, the longer half-life of QDs@CMPs maintained a relatively high level of these molecules in circulation, which was required for EPR-based cancer-targeting efficiency. Second, integrins on the CMPs surface might interact with their ligands on cancer cells, resulting in their active targeting ability. Based on these results, the author loaded both a survivin siRNA and Ag_2_Se@Mn QDs into CMPs (survivin siRNA@QDs@CMPs) via electroporation to establish a dual-modality traceable nanoplatform, which substantially inhibited tumor growth without causing weight loss in vivo.

#### Metallic materials in combination with other treatments

*With photodynamic therapy* Photodynamic therapy is a technique that utilizes the photodynamic effects of drugs to treat diseases [144]. Its therapeutic components include photosensitizers, specific wavelengths of light, and oxygen [39]. After the uptake of the photosensitizer by cells, the photosensitizer is excited by a laser with a specific wavelength and then changes from the ground state to the triplet excited state. Triplet excited photosensitizers react directly with substrates such as the cell membrane or some biological macromolecules to form free radicals that kill cells. These molecules also transfer energy to surrounding oxygen to produce ROS. On one hand, ROS cause acute damage to microvessels in tumors, resulting in vascular obstruction and ischemia. On the other hand, they directly cause the death of tumor cells to achieve the purpose of local treatment of tumors. Amanda et al*.* incubated THP-1 macrophages with 8 nm citrate-coated iron oxide nanoparticles (CCIONs) and m-THPC, a clinically used photosensitizer, and then starved them for 2 days to elicit the release of CCION and m-THPC-packaging and macrophage-derived MPs (CCION/m-THPC@M-MPs) [145]. After a short centrifugation to remove cells and apoptotic bodies, the medium was placed in a magnetic field gradient of 55 Tm^−1^ for CCION/m-THPC@M-MPs purification. Characterization and detection showed that CCION/m-THPC@M-MPs had a mean vesicle size of 332 ± 94 nm and emitted red fluorescence for 2 months when stored at 4 °C and 6 months at −20 °C. CCION/m-THPC@M-MPs were intratumorally injected into solid subcutaneous TC-1 tumors to determine their imaging function. A large area with a hypointense signal was observed in MRI scans of tumors, and the fluorescence signal observed upon excitation with blue light was observed 20 h after injection. A potential explanation for this finding is that the m-THPC spreads toward the tumor surface. In addition to the imaging capacity, CCION/m-THPC@M-MPs plus light exposure (λ = 630 nm, 30 J/cm^2^, 77 mW/cm^2^ for 390 s) also exhibited significant anticancer efficacy. The weakness of this study was that it did not examine the biosafety of CCION/m-THPC@M-MPs in vivo.

*With immune checkpoint therapy* Recently, immune checkpoint blockade (ICB) has become an essential strategy in oncotherapy. However, the low response rate (approximately 20%) in clinical practice has prompted the development of new methods to improve its success [146]. In this regard, promoting immunogenic cell death in cancer cells became a promising strategy for cancer treatment [147]. Zhang et al. designed an MPs-based delivery system to enhance the efficacy of immunotherapy through packaging with metallic materials and surface engineering [148]. Considering their strong M1-like polarization capacity in macrophages [149–151], nano-Fe_3_O_4_ was incubated with tumor cells for 16 h, followed by UV irradiation to elicit Fe_3_O_4_@UT-MPs. Furthermore, CpG, a potent Toll-like receptor 9 agonist [152], was packaged into liposomes (CpG@Lipo), which were subsequently tethered on the surface of Fe_3_O_4_@UT-MPs through maleimide-sulfhydryl chemical linkage to stimulate a more powerful immune response, thus forming CpG@Fe_3_O_4_@UT-MPs. As expected, CpG@Fe_3_O_4_@UT-MPs was verified to transform TAMs into an antitumor phenotype, enhance DCs maturation, augment CTL and Th cell infiltration and increase the secretion of immunostimulatory cytokines, such as TNF-α, IL-12 and IFN-γ, both in vivo and in vitro. By altering “cold” tumors into “hot” tumors, CpG@Fe_3_O_4_@UT-MPs was capable of inhibiting cancer growth, prolonging mouse survival, and enhancing the therapeutic effect of the anti-PD-L1 antibody, representing an effective approach to improve ICB.

#### Multiple PEG-based methods for combination therapy

*Chemotherapy and targeted therapy* Because 1,2-dioleoyl-sn-glycero-3-phosphoethanolamine-poly (ethylene glycol) (DSPE-PEG), which has been approved by the FDA for clinical applications and self-assembles into lipid bilayers, Chen et al*.* exploited this characteristic and engineered macrophage-derived MPs (M-MPs) as a new DDS [153]. Based on increased expression of folate (FA) receptors between cancer cells (over) and normal cells (limited) [154], macrophages were cultured in medium containing FA-functionalized DSPE-PEG (DSPE-PEG-FA) and biotin-functionalized DSPE-PEG (DSPE-PEG-Biotin) for 48 h and then were starved for another 48 h to allow the release of biotin and FA-modified MPs (FA/biotin@M-MPs). Streptavidin (SA)-conjugated iron oxide nanoparticles (SA-IONPs) were added to the supernatant to form FA/IONP@M-MPs and conveniently and rapidly isolate FA/biotin@M-MPs, which were simply separated with a magnet (100 × 50 × 20 mm, 0.6 T). The superficial FA and IONPs on M-MPs endowed M-MPs with a remarkable targeting capacity in tumor-bearing mice without causing any significant abnormalities in liver and kidney functions, hemoglobin or the histology of major organs. Furthermore, after encapsulating DOX into FA/IONP@M-MPs through electroporation at 250 V and 350 μF, the newly formed DOX@FA/IONP@M-MPs displayed striking antitumor activity after intravenous injection in a HeLa xenograft-bearing mouse model.

*Chemotherapy and siRNA therapy* Similarly, Zhang et al*.* designed another type of FA-modified MPs with SA-QD modification on the surface [155]. Tumor cells were cultured in medium containing 1% DSPE-PEG-biotin and 1% DSPE-PEG-FA and starved for 2 days. Afterward, FA/biotin@T-MPs were collected through centrifugation. Flow cytometry technology showed that the modification efficiencies of FA and biotin were 54.9% and 60.2%, respectively. Confocal scanning microscopy revealed that FA/biotin@T-MPs entered the cytoplasm of cancer cells through endocytosis instead of adsorbing on the cell surface, thus guaranteeing efficient drug delivery. Considering the synergistic effect of a Bcl-2 siRNA (Bcl-2, anti-apoptotic protein) and paclitaxel (a typical chemotherapy drug to destroy microtubules) (Taxol) [156, 157], the authors packaged them together into FA/biotin@T-MPs via electroporation at 250 V and 350 μF, forming a new DDS (Bcl-2 siRNA/Taxol@FA/biotin@T-MPs). Tumor cells treated with Bcl-2 siRNA/Taxol@FA/biotin@T-MPs showed the greatest inhibition of Bcl-2 expression and microtubule damage, leading to the strongest toxicity both in vitro and in vivo. This effect might be explained by the increased target capacity attributed to FA, with the anticancer efficacy decreasing up to 15.1% for Bcl-2 siRNA/Taxol@biotin@T-MPs treatment. Furthermore, for in vivo tumor imaging, FA/biotin@T-MPs modified with SA-QDs (CdSe/ZnS) (FA/QD@T-MPs) also produced good results. However, although the biocompatibility of Bcl-2 siRNA/Taxol@FA/biotin@T-MPs was detected and verified to be high, it was not explored in FA/QD@T-MPs treatment, and the therapeutic effect of FA/QD@T-MPs loaded with the Bcl-2 siRNA and Taxol was also not evaluated.

*Targeted therapy and immune checkpoint therapy* Considering the immunosuppressive function of TAMs, treatments targeting TAMs have become a strategy for improving antitumor immunity. Recently, Yang et al*.* developed a new platform to target TAMs and reprogram to the M1 phenotype [158], in which macrophages were chosen as the parental cells for MPs due to the tumor-targeting capacity of M-MPs. Because TAMs express the mannose (Man) receptor CD206 at high levels [159], MPs released from DSPE-PEG-Man-incubated macrophages are also Man-modified (Man@M-MPs) and show a selective TAM targeting capacity. Furthermore, metformin (Met), a popular drug used to treat diabetes, was incubated with Man-engineered macrophages, followed by UV irradiation to obtain Met-packaged Man@UM-MPs (Met@Man@UM-MPs) and repolarize TAMs to the M1 phenotype. In this study, Met@Man@UM-MPs injected intravenously accumulated in tumor tissues and were mainly enriched in TAMs compared with many other types of immune cells. In addition, Met@Man@UM-MPs -stimulated TAMs repolarized to the M1 phenotype through an adenosine 5′-monophosphate (AMP)-activated protein kinase signaling pathway and reduced the viability of tumor cells by secreting multiple TNF-α molecules and increasing the phagocytosis ability. Moreover, with the help of MMP9 and MMP14 in M-MPs, collagen I in the extracellular matrix was degraded, thus transforming the hard tumor parenchyma into soft tissue. Afterward, the infiltration of immune cells and other drugs into the tumor tissues was substantially increased. Twenty-four hours after the injection of Met@Man@UM-MPs, the anti-PD-1 drug significantly accumulated in tumor tissues and cooperated with Met@Man@UM-MPs to exert antitumor activity.

## Prospects, challenges and conclusions

In the past decade, research on MPs for cancer treatments has increased substantially [160]. The various characteristics of MPs, such as size, surface charge and compositions, make them successful in terms of utility for inducing antitumor activity. When used as therapeutic drugs, MPs inhibit tumor growth by directly killing and/or activating antitumor immunity. When functioning as drug carriers, MPs effectively deliver cargoes to tumor cells (and even into their nuclei) without causing serious side effects. These exciting studies make MPs a new approach in oncotherapy.

For clinical translation of MPs-based therapeutics, many questions remain to be explored and answered. (1) The first one is about the preparation of therapeutic MPs, which includes three aspects to be considered. The first one is the choice of cell source for MPs. One option is to collect MPs from autologous human blood samples, which takes advantages of convenience without immune rejection. Another option is the autologous tumor cells, whose MPs carry a large number of tumor antigens to activate specific antitumor immunity, hence achieving individualized treatment. However, both of these two methods have the problem of limited MPs production and are not suitable for large-scale batch preparations. To address this issue, some scientists raised the usage of cell membranes from tumor cell [161], which are abundant and could be prepared into the same size of MPs. However, this method also led to the loss of inner compositions of MPs which may have biological functional. The other way is to use the stable human cell lines, which quickly proliferate and can be the source for large amounts of MPs. However, their immunogenicity may become a question, which is an issue that needs more study. The second aspect is the type and intensity of stimulus applied to parental cells, which also require extensive screening and comparison. As listed in Table 2, these two factors influence the biological functions of MPs. The third factor is the selection of modification method and combination with existing therapeutic regimens. Since, there are many modification methods to enhance the functionality of MPs (Fig. 2), as well as many therapies for which MPs can mediate synergistic or combination treatment effects (Fig. 4). (2) Another critical question is the large-scale pharmaceutical production of MPs, which is the foundation for large clinical trials. Therefore, standardized isolation techniques and storage conditions are important to ensure reproducibility, safety and long-term stability. Furthermore, it is difficult to obtain reproducible and uniform size of MPs, which is also an important factor hindering their clinical application. Purification technology based on modern microfluidics may help solve this problem. But these will take pharmaceutical development efforts and expertise to achieve. (3) Furthermore, the administration route is also an essential question for the therapeutic effect and indication selection. Chemo@T-MPs have been used in clinical trials with local injections (Table 5). Although the safety of chemo@T-MPs intravenous administration has been established in mice, it has not been demonstrated yet in humans. The clearance of chemo@T-MPs mediated by the reticular phagocytosis system may be a critical issue, which decreases the accumulation of chemo@T-MPs in tumors and limits efficacy. Moreover, at the same time, the reticular phagocytic system may be damaged during the clearance, leading to side effects. The introduction of CD47 expression on T-MPs may be helpful to avoid clearance since CD47 delivers a “do not eat me signal” to macrophages though binding to their signal-regulatory protein alpha (SIRPα) receptors [162]. However, this also risks mediating immune escape by transferring CD47 to tumor cells. Therefore, the safety and efficacy of modified-MPs with intravenous administration in cancer treatment requires clinical study. (4) Finally, the most important issue for clinical translation of MPs-based therapeutics is clinical trials. Considering the great differences between model animals and human beings, clinical trials are essential for establishing the merit of MPs and validation of disease indications.

In summary, with increasing in-depth research on MPs, their use in applications for treating cancers has attracted more attention. There is no doubt that MPs have compelling advantageous features both as therapeutic agents and as drug delivery carriers. However, there are also many obstacles impeding transition of MPs-based therapeutics. Further fundamental and translational research on MPs is still required, with more clinical trials ultimately needed for validation of their utility for human treatments.

## Data Availability

Not applicable.

## Abbreviations

MPs: Microparticles
EVs: Extracellular vesicles
ER: Endoplasmic reticulum
ATP: Adenosine triphosphate
ARF: ADP-ribosylation factor
MLC: Myosin light-chain
ROCK: RHO-associated protein kinase
LIMK: Lim kinase
ERK: Extracellular signal-regulated kinase
MLCP: Myosin light chain phosphatase
PLD: Phospholipase D
MLCK: MLC kinase
MAPK: Mitogen-activated protein kinase
NF-κB: Nuclear factor kappa-B
PAR: Protease-activated receptor
TNF-α: Tumor necrosis factor-α
EPR: Enhanced permeability and retention
TSG101: Tumor susceptibility gene 101 protein
UV: Ultraviolet
IL: Interleukin
TLR3: Toll-like receptor 3
UT-MPs: Ultraviolet-induced and tumor-derived microparticles
RT-MPs: Radiation-induced and tumor-derived microparticles
MPE: Malignant pleural effusion
ROS: Reactive oxygen species
GSH: Glutathione
TME: Tumor microenvironment
TAMs: Tumor-associated macrophages
PD-L1: Programmed cell death ligand 1
MDSCs: Myeloid-derived suppressor cells
PAMPs: Pathogen-associated molecular patterns
iNOS: Inducible nitric oxide synthase
TRPML2: Mucolipin 2
DCs: Dendritic cells
IFN: Type I interferon
NOX2: NADPH oxidase 2
MHC: Major histocompatibility complex
TAP: Antigen peptide transporter
TFEB: Transcription factor EB
RILP: Rab interacting lysosomal protein
TRCs: Tumor-repopulating cells
IECs: Intestinal epithelial cells
NOD2: Nucleotide binding oligomerization domain 2
NMIBC: Nonmuscle-invasive bladder cancer
LAMP: Lysosomal associated membrane protein
MTX: Methotrexate
DOX: Doxorubicin
P-gp: P-glycoprotein
PMNs: Neutrophils
NETs: Neutrophil extracellular traps
CCA: Cholangiocarcinoma
UDPG: Nucleotide sugar uridine diphosphoglucose
NLRP3: NOD-like receptor family, pyrin domain containing 3
ASC: Apoptosis-associated speck-like protein containing C-terminal caspase recruitment domain
cGAS: Cyclic GMP-AMP synthase
STING: Stimulator of interferon genes
GSDME: Gasdermin E
MMP8: Matrix metalloproteinase 8
NO: Nitric oxide
FDA: Food and Drug Administration
MSCs: Mesenchymal stem cells
OVs: Oncolytic virus
CAR: Coxsackie-adenovirus receptor
CSCs: Cancer stem cells
pDNA: Plasmid DNA
TK: Thymidine kinase
NTR: Nitroreductase
VSV-G: Vesicular stomatitis virus G protein
AMNPs: Anionic citrate-coated Fe_3_O_4_ nanoparticles
E-MPs: Endothelial cell-derived microparticles
MRI: Magnetic resonance imaging
HUVECs: Human umbilical vascular endothelial cells
QDs: Quantum dots
AuNPs: Gold nanoparticles
IONPs: Iron oxide nanoparticles
IONCs: Iron oxide nanocubes
Au/IONPs: Gold/iron oxide heterodimers
GNS: Gold nanostar
DDS: Drug delivery system
PA: Photoacoustic
CT: Computed tomography
VEGF: Vascular endothelial growth factor
NIR: Inear infrared ray
SA: Streptavidin
CMPs: Circulating microparticles
CCIONs: Itrate-coated iron oxide nanoparticles
ICB: Immune checkpoint blockade
Lipo: Liposomes
DSPE-PEG: 1, 2-dioleoyl-sn-glycero-3-phosphoethanolamine-poly: Ethylene glycol
M-MPs: Macrophage-derived microparticles
FA: Folate
Taxol: Paclitaxel
Man: Mannose
Met: Metformin
AMP: Adenosine 5′-monophosphate
SIRPα: Signal-regulatory protein alpha
CAFs: Carcinoma-associated fibroblasts
Lm: Listeria monocytogenes
ICAM-1: Intercellular adhesion molecule-1
LPS: Lipopolysaccharide
PMA: Phorbol 12-myristate 13-acetate
